# Protease activated receptor 1 (PAR1) enhances Src-mediated tyrosine phosphorylation of NMDA receptor in intracerebral hemorrhage (ICH)

**DOI:** 10.1038/srep29246

**Published:** 2016-07-07

**Authors:** Zhen-Zhen Duan, Feng Zhang, Feng-Ying Li, Yi-Fei Luan, Peng Guo, Yi-Hang Li, Yong Liu, Su-Hua Qi

**Affiliations:** 1 Research Center for Biochemistry and Molecular Biology and Provincial Key Laboratory of Brain Disease Bioinformation, Xuzhou Medical College, Xuzhou, 221002, P. R. China

## Abstract

It has been demonstrated that Src could modulate NMDA receptor, and PAR1 could also affect NMDAR signaling. However, whether PAR1 could regulate NMDAR through Src under ICH has not yet been investigated. In this study, we demonstrated the role of Src-PSD95-GluN2A signaling cascades in rat ICH model and *in vitro* thrombin challenged model. Using the PAR1 agonist SFLLR, antagonist RLLFS and Src inhibitor PP2, electrophysiological analysis showed that PAR1 regulated NMDA-induced whole-cell currents (*I*_NMDA_) though Src in primary cultured neurons. Both *in vivo* and *in vitro* results showed the elevated phosphorylation of tyrosine in Src and GluN2A and enhanced interaction of the Src-PSD95-GluN2A under model conditions. Treatment with the PAR1 antagonist RLLFS, AS-PSD95 (Antisense oligonucleotide against PSD95) and Src inhibitor PP2 inhibited the interaction among Src-PSD95-GluN2A, and p-Src, p-GluN2A. Co-application of SFLLR and AS-PSD95, PP2, or MK801 (NMDAR inhibitor) abolished the effect of SF. In conclusion, our results demonstrated that activated thrombin receptor PAR1 induced Src activation, enhanced the interaction among Src-PSD95-GluN2A signaling modules, and up-regulated GluN2A phosphorylation after ICH injury. Elucidation of such signaling cascades would possibly provide novel targets for ICH treatment.

Thrombin, a serine protease generated by the cleavage of prothrombin, is an essential component of the coagulation cascade. It is produced in the brain either immediately after cerebral hemorrhage or after the blood-brain barrier (BBB) breakdown that occurs following brain injuries^1^. It has been demonstrated that the role of thrombin acting on its receptors in ischemic, hemorrhagic and traumatic brain injury is concentration-dependent. Increasing evidence indicates that high concentrations (more than 500 nM) of thrombin within the brain parenchyma can be deleterious^2^,^3^. For example, thrombin can induce brain edema, thrombosis and inflammation by directly acting on its receptors, protease activated receptors (PARs)^4^,^5^. PARs are a subfamily of G protein-coupled receptors (GPCRs) with four members, namely, PAR1, PAR2, PAR3 and PAR4. PAR1 is highly expressed in many different cell types and transmits signals by coupling with Gαq/11, Gαi/O and Gα12/13 proteins^6^,^7^. Under physiological condition, PAR1 is detected in the mammalian brain cortex, basal ganglia, and striatum and nucleus accumbens neurons. PAR1 plays an important role in astrocyte proliferation, stimulus induced long-term potentiation (LTP) and nerve growth factor (NGF) secretion^8^,^9^,^10^. Activation of PAR1 by thrombin at high concentrations contributes to the transient focal cerebral ischemia injury^11^. In addition, PAR1 deficient mice had a 3.1-fold reduction in infarct volume after transient focal cerebral ischemia. Similarly, intracerebro-ventricular injection of PAR1 antagonist BMS-200261 reduced infarct volume by 2.7-fold^11^. These observations suggest that PAR1 activation exacerbates neuronal damage in many brain injury models in a manner that is dependent on thrombin overproduction^12^.

The N-methyl-D-aspartic acid receptor (NMDAR) is a ligand-gated ion channel receptor composed of two types of subunits, GluN1 and GluN2 subunits^13^. NMDAR plays a critical role in many pathological processes of brain injuries and has been implicated in neurological diseases such as stroke, traumatic brain injury, dementia and schizophrenia^14^. Under ischemia and reperfusion (I/R) condition, excessive glutamate release causes influx of Na^+^ and Ca^2+^ through the NMDAR^15^,^16^. Activation of Src kinases by influx of Ca^2+^ mediates GluN2A and GluN2B tyrosine phosphorylation^17^, up-regulates NMDAR activity, feedback-increases the influx of Ca^2+^ and aggravates the brain damage^18^,^19^. Previously we reported that cerebral I/R could induce Src autophosphorylation and activation, resulting in tyrosine phosphorylation and activation of NMDA receptor, and that postsynaptic density protein 95 (PSD95) participated in the formation of Src-PSD95-GluN2A signaling modules^19^,^20^. Although Src is known to bind to PSD95 and mediate phosphorylation of NMDA receptors^21^, how Src participates in NMDAR activation in ICH model is unclear yet.

It has been reported that PAR1 activation led to cell depolarization and potentiation of synaptically activated NMDAR function in dentate granule neurons of hippocampus^12^. Indeed, Shigetomi *et al*. found that the NMDAR was involved in PAR1 mediated Ca^2+^ elevation via PAR1-NMDA interplay in astrocytes^22^. Numerous studies suggest that PAR1 can enhance neuronal excitability associated with NMDAR mediated neuronal damage and NMDAR activation is necessary for thrombin/PAR1-induced neurodegenerative effects under pathological conditions, such as cerebral ischemia and hemorrhage^22^,^23^,^24^,^25^. The route from PAR1 activation to NMDA receptor current potentiation has been described in ischemia by the Traynelis group^23^, however, the underlying mechanism by which PAR1 affects NMDAR and induces brain injuries under ICH condition remains to be addressed.

In this study, we tested our hypothesis that activation of PAR1 by thrombin during ICH enhanced interaction of Src-PSD95-GluN2A signaling modules and increased the phosphorylation of Src and NMDAR, which eventually led to brain injuries. To determine the mechanism of PAR1 in the regulation of NMDAR during ICH, we used PAR1 agonist SFLLR and antagonist RLLFS, antisense oligonucleotide against PSD95 AS-ODNs, Src antagonist PP2 or NMDAR antagonist MK-801. We expect that elucidation of PAR1 signaling pathway in ICH would provide potential drug targets and strategies for treatment of hemorrhagic brain injuries.

## Experimental Procedures

### Antibodies, peptides and reagents

GluN2A (AB1555, 1:1000) was purchased from Millipore (Billerica, MA, USA). Goat anti-rabbit IgG-AP and goat anti-mouse IgG-AP antibodies were purchased from Sigma-Aldrich Corp. (St. Louis, MO, USA) while antibodies for Src (ab7950, 1:500), phosphotyrosine (ab9319, 1:500) and anti-β-actin antibody (ab32572) were purchased from Abcam Biotechnology (Cambridge, UK). Anti-Phospho-Src Family (Tyr416) (2101S, 1:1000), anti-PSD95 (2507S, 1:1000) were obtained from Cell Signaling Biotechnology (Beverly, MA, USA). Peptides including SFLLR (SF), RLLFS (RL), AS and MS- PSD95 were obtained from Shanghai Biotechnology Company (Shanghai, China). The sequences of antisense and missense ODNs against PSD95 are as follows: AS, 5′-TGTGATCTCCTCATACTC-3′; and MS, 5′-AAGCCCTTGTTCCCATTT-3′.

### Experimental cerebral hemorrhagic model

One hundred and fifty adult male Sprague-Dawley (SD) rats weighing 200–250 g were obtained from Shanghai Experimental Animal Center (Shanghai, China). The experimental procedures and protocols were approved by the Animal Ethics Committee of Xuzhou Medical College (Approval ID: SCXK (SU) 2010-0003). All experiments were conducted in compliance with institutional guidelines. The animals were randomly divided into control group, in which rats did not receive any treatment; ICH group, in which rats were subjected to ICH; dissolvent group, in which rats were intraperitoneally or intracerebro-ventricularly given corresponding solvent, H_2_O, 0.9% NaCl, 10 mM TE buffer or 1% DMSO; drug treatment group, in which rats subjected to ICH were treated with SF, RL, SF + RL, MS, AS, SF + AS, PP2, SF + PP2, MK801 respectively. ICH model was developed as previously described^26^. Briefly, rats were intraperitoneal injected with 10% chloral hydrate (300 mg/kg) for anesthesia. After anesthesia induction, rats were fixed at prone position on the stereotaxic apparatus. Subsequently, 50 μl of autologous whole blood was injected into the right caudate nucleus (striatum coordinates: 1.0 mm anterior, 3.0 mm lateral and 5.0 mm ventral, with respect to Bregma). Rats were allowed for free access to food and water when the incision was sutured. To confirm the success of the model, nerve functional scores tests were conducted according to Zea Longa *et al*.^27^. The nerve function was scored on a five-point scale: a score of 0 indicated no neurologic deficit, a score of 1 (failure to extend left forepaw fully) a mild focal neurologic deficit, a score of 2 (circling to the left) a moderate focal neurologic deficit, and a score of 3 (falling to the left) a severe focal deficit; rats with a score of 4 did not walk spontaneously and had a depressed level of consciousness. Level 2 to 4 were considered as successful model.

SFLLR (SF, 300 μM) was administered 30 min before hemorrhage and RLLFS (100 μM) was administered 30 min after hemorrhage (at the dose of 10 μl) in rats via intracerebroventricularly injection. One hundred micrograms of specific antisense ODNs against PSD95 (AS-PSD95) in 10 μl TE buffer (10 mM Tris-HCl, pH 8.0, 1 mM EDTA) were administrated to the rats every 24 hr for 3 days through cerebral ventricular injection before ICH (IV, anteroposterior, 0.8 mm; lateral, 1.5 mm; depth, 3.5 mm from bregma). The same dose of missense ODNs (MS-PSD95) and vehicle were used as control. MK-801 (3.0 mg/kg) was given twice injection 30 min and 60 min before hemorrhage by IP. Ten microliters of PP2 (15 μg dissolved in DMSO to make 10% solution) was injected 30 min before hemorrhage by IV. In parallel experiments, solvent was used as control.

### Sample preparation

After 3 h of ICH, the rats were decapitated and the brain caudate putamen was obtained. The brain tissues were added to 1.0 ml of homogenization buffer and homogenized using Teflon homogenizer (10 s × 6 times). Following the addition of 1.0 ml homogenization buffer, the samples were centrifuged at 2000 g at 4 °C for 20 min, and the supernatant was collected (mainly the caudate putamen cytoplasmic portion). Protein concentration was determined by the Lowry method.

### Immunoprecipitation and immunoblotting

For immunoprecipitation, tissue homogenates (400 μg of protein) were diluted four-fold with HEPES buffer containing 50 mM HEPES (pH 7.4), 150 mM NaCl, 10% glycerol, 1% Triton X-100, and 1 mM each of EGTA, EDTA, PMSF and Na_3_VO_4_. Samples (from ICH models) were pre-incubated for 1 h with 20 μl protein A/G and then centrifuged to remove any adhered protein. The supernatants were incubated with 2–5 μg proper antibody for 4 h at 4 °C. After the addition of proteinA/G-sepharose, the mixture was incubated at 4 °C for 2 h. After being washed with HEPES buffer, samples were eluted by sodium dodecyl sulfate-polyacrylamide gel electrophoresis (SDS-PAGE) loading buffer and boiled at 100 °C for 5 min. Supernatants were used for western blot analysis.

Western blot analysis was carried out on 10% SDS-PAGE gel. Proteins (100 μg) were electro-transferred onto nitrocellulose filter (NC, pore size, 0.45 μm). After blocking for 3 h in phosphate buffer (PBS) with 0.1% Tween 20 (PBST) and 3% BSA, the membranes were incubated overnight with primary antibody in PBST containing 3% BSA. Detection was carried out by using proper alkaline phosphatase conjugated IgG (1:20,000) and developed with NBT/BCIP assay kit (Promega, Madison, USA).

### Primary neuronal cell culture

Primary cultured hippocampal neurons were prepared from 18-day-old SD rat embryos. In brief, hippocampi were isolated in ice-cold high-glucose Dulbecco’s modified Eagle medium (Gibco, Grand Island, NY). Hippocampal cells were dissociated by trypsinization [0.25% (wt/vol) trypsin and 0.02% (wt/vol) EDTA in Ca^2+^ and Mg^2+^ -free Hank’s balanced salt solution] at 37 °C for 15 minutes, followed by gentle triturating in plating medium (high-glucose Dulbecco’s modified Eagle medium supplemented with 10% fetal bovine serum and 10% horse serum; Gibco). Cells were seeded onto poly-L-lysine (Sigma)-coated wells or cover slips at a density of 0.5 × 10^5^ cells/cm^2^ and incubated at 37 °C in 5% CO_2_ atmosphere. After 24 hours, culture medium was replaced with neurobasal medium supplemented with B-27 (Gibco) and 0.5 mM glutamine. Medium was half-replaced twice every week.

### Patch Clamp Recording

All experiments were carried out at room temperature (22 ~ 25 °C). Electrophysiological recording was performed in the conventional whole-cell patch-clamp recording configuration under voltage-clamp conditions as previous reported^28^. Briefly, glass pipettes were pulled from 1.5 mm glass capillaries on a two-stage puller (PP-830, Narishige). The resistance between the recording electrode filled with pipette solution (in mM: CsCl 140, TEA 20, Hepes 10, EGTA 5, MgCl_2_ 1, Mg-ATP 4, pH7.2) and the reference electrode was 3–5 MΩ in standard extracellular solution (SS, in mM: NaCl 140, glucose 10, Hepes 10, KCl 5, CaCl_2_ 2, MgCl_2_ 1, pH7.4). Membrane currents were detected with AxonPatch 700B (Axon Instruments, Foster City, CA, USA), filtered at 2 kHz, sampled and analyzed using a DigiData 1440A interface and a computer with the pClamp 10 system (Axon Instruments). The series resistance compensation was automated. The membrane potential was held at −60 mV throughout the experiment. NMDA receptor-mediated whole-cell currents (*I*_NMDA_) was detected by application of 100 μmol/L NMDA dissolved in Mg^2+^ -free SS following 5 s application of Mg^2+^ -free SS. Src kinase inhibitor PP2 and its non-active analog PP3 were administered in the pipette solution. The *I*_NMDA_ were detected at least 5 min after the cells were patched to allow the diffusion of PP2 and PP3. The chemical reagents used in patch-clamp recording were obtained from Sigma-Aldrich except otherwise indicated.

### COS7 Cell Culture, transfection and treatments

COS7 Cells derived from monkey kidney were grown in Dulbecco’s modified Eagle medium (DMEM; Gibco BRL, Grand Island, NY) supplemented with 10% fetal calf serum (37 °C, 5% CO_2_) in 5 cm × 5 cm or 5 cm × 7 cm culture flasks in a humidified incubator. Cells were transfected with the indicated plasmid vectors (PSD95, 6 μg; 6 μg GluN2A; or 3 μg Src) at the time of 90–95% confluent by using Lipofectamine™ 2000. COS7 cells were incubated for forty eight hours and were stimulated with thrombin (5 U/ml) for 1 hour to mimic the ICH model followed by immediate harvest. PP2 (10 μmol/L) or control buffer was added to cells 30 minute before thrombin treatment, while SF (1 μmol/L) was added to cells 60 minute before thrombin treatment.

### Statistical Analysis Statistical

Statistical analysis was carried out by using one-way ANOVA followed by Newman-Keuls test. *P* < 0.05 was considered as statistically significant. Data are expressed as Mean ± S.D.

## Results

### Activated PAR1 up-regulated NMDA receptor ion channel activity via Src kinase in primary cell cultures

**Figure 1:**
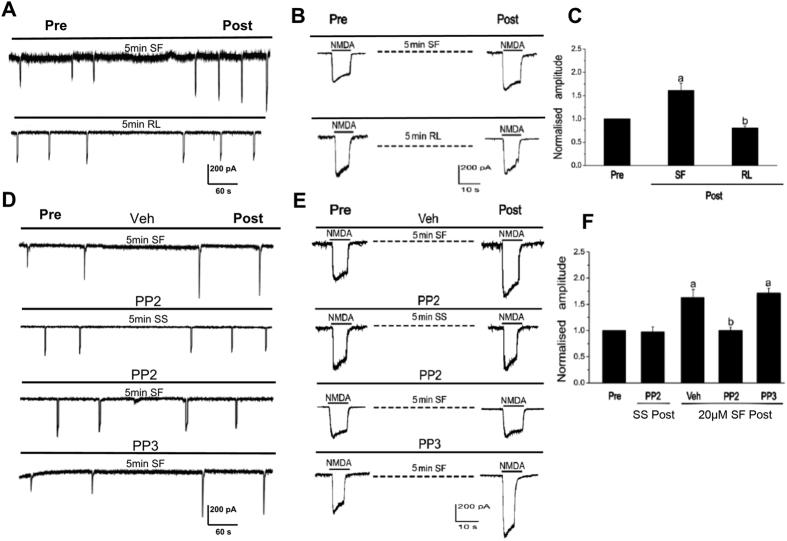
PAR1 up-regulated NMDA receptor ion channel activity via Src kinase. (**A,B**) PAR1 agonist SF and antagonist RL affected NMDA receptor-mediated whole-cell currents (*I*_NMDA_) on 12- to 16 day cultured rat hippocampal neurons. (**C**) Statistics for *I*_NMDA_ after stimulation for 5 min by SF or RL (^a^*P* < 0.05 vs. group of pre-Control, ^b^*P* < 0.05 vs group of SF, n = 6 from 3 cultures). (**D,E**) The effects of Src family kinase inhibitor PP2 and its non-active analog PP3 on *I*_NMDA_. (**F**) Statistic results of effects of PP2 and PP3 on SF upregulation effects on I_*NMDA*_ (^a^*P* < 0.05 vs. group of SS + PP2, ^b^*P* < 0.05 vs. group of SF + Vehicle, n = 6 from 3 cultures).

To investigate the interactions of PAR1 with NMDA receptors, NMDA receptor-mediated whole-cell currents (*I*_NMDA_) were recorded by patch-clamp recording on 12- to 16-day cultured rat hippocampal neurons. As shown in Fig. 1A–F, 5 min incubation of PAR1 agonist SF (20 μmol/L) could lead to an increase in *I*_NMDA_, while incubation of PAR1 antagonist RL led to a decrease of *I*_NMDA_. To further investigate the signaling mechanism of the upregulation effects of SF on *I*_NMDA_, 2 μmol/L Src family kinase inhibitor PP2 and its non-active analog PP3 were used through intracellular administration. *I*_NMDA_ was detected at least 5 min after the cells were patched. As shown in Fig. 1C,D, intra-pipette administration of PP2 or PP3 did not affect the basal *I*_NMDA_. However, the upregulation effects of SF on *I*_NMDA_ were blocked by PP2.

### PAR1 activated Src-mediated phosphorylation of NMDAR after ICH

**Figure 2:**
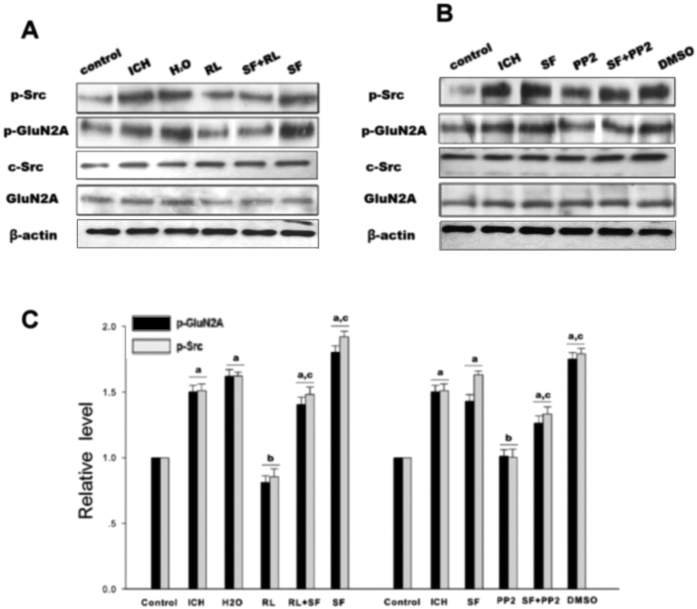
PAR1 activated Src-mediated phosphorylation of NMDAR after rat ICH in caudate putamen (CPu). (**A**) p-Src, c-Src, GluN2A were determined by western blot analysis and p-GluN2A was examined by immunoprecipitation with anti-p-Y antibody followed by immunoblotting with antibody against NR2A in control, ICH, H_2_O, RL, SF + RL and SF groups. β-actin was used as a loading control. (**B**) Similar to A, but in control, ICH, SF, PP2, SF + RL and DMSO groups. (**C**) Statistic the results of p-Src and p-GluN2A in each experimental group. The results are shown as mean ± S.D (n = 5). ^a^*P* < 0.05 vs. Control group, ^b^*P* < 0.05 vs. ICH 3 h treatment group, ^c^*P* < 0.05 vs. RL-treatment group.

To study PAR1 induced phosphorylation of NMDAR via Src in ICH model, rats were treated with the PAR1 agonist SFRRL, antagonist RLLSF or Src inhibitor PP2. Results showed that phosphorylation of Src and GluN2A were significantly increased in the hemorrhagic brain compared with the control rats. RLLSF reduced the p-Src and p-GluN2A compared with the ICH, SF and H_2_O groups (H_2_O, water, the solvent of SF or RL). To further prove PAR1 regulation of the NMDAR function, co-application of RL and SF, it was found that RL + SF neutralized the effect of SF on p-Src and p-GluN2A (Fig. 2A,C). These results indicated the important role of PAR1 in the regulation of Src and NMDAR phosphorylation after ICH. To further demonstrate that PAR1 regulates the NMDAR function though Src, we treated the rats with Src inhibitor PP2. Results showed that PP2 treatment significantly reduced the phosphorylation of Src and GluN2A when compared with either ICH or SF or DMSO (DMSO, the solvent of PP2) groups. Additionally, we also found that when Src inhibitor PP2 and PAR1 agonist SF were combined, the inhibitory role of PP2 was abolished and phosphorylation of Src and NR2A was enhanced (Fig. 2B,C). The total protein of GluN2A and Src remained unchanged.

**Figure 3:**
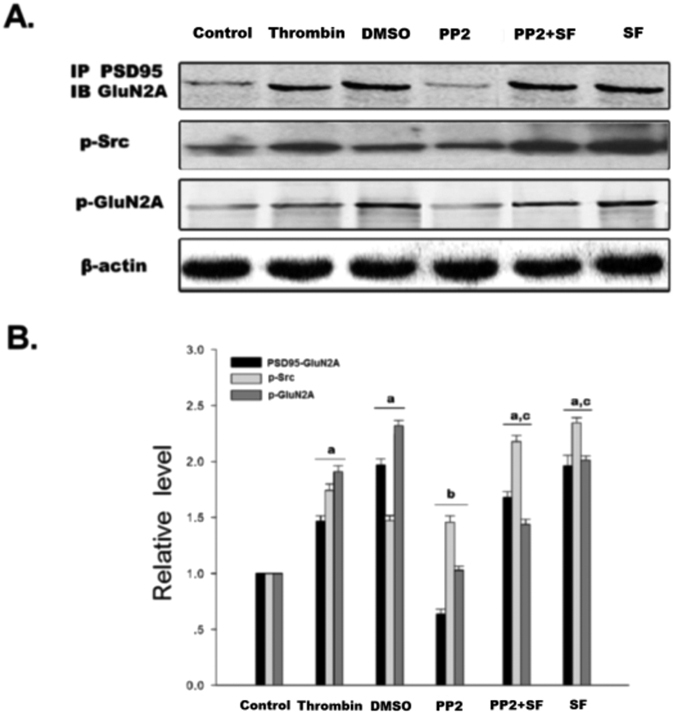
Thrombin enhanced the interaction of PSD95 and GluN2A, as well as phosphorylation of Src and GluN2A in COS7 cells. (**A**) The interaction of PSD95 and GluN2A was examined by immunoprecipitation (IP) with PSD95 antibody followed by immunoblotting with anti-GluN2A antibody. The expression level p-Src was detected by immunoblotting with anti- p-Src antibody. Tyrosine phosphorylation of GluN2A was detected by immunoprecipitation of p-Y and immunoblotting of GluN2A. β-actin was used as a loading control. (**B**) Statistics for the interaction of PSD95 and GluN2A, p-Src and p-GluN2A in control, thrombin, DMSO, PP2, PP2 + SF and SF groups. The results are shown as mean ± S.D (n = 5). ^a^*P* < 0.05 vs. Control, ^b^*P* < 0.05 vs. Thrombin treatment group, ^c^*P* < 0.05 vs. PP2- treatment group.

*In vitro*, treatment of COS7 cells with thrombin (5 U/ml) enhanced the phosphorylation of Src and GluN2A when compared with the control group. PP2 treatment significantly reduced the p-Src and p-GluN2A when compared with thrombin or DMSO (the solvent of PP2) groups. When Src inhibitor PP2 and PAR1 agonist SF were combined, the inhibitory role of PP2 was reversed. p-Src and p-GluN2A were enhanced in PP2 + SF groups when compared with the PP2 group. The total protein of GluN2A and Src remained unchanged (Fig. 3A,B). Both *in vivo* and *in vitro* results suggested that PAR1 mediated phosphorylation of GluN2A via Src.

### ICH enhanced the assembly of the Src-PSD95-GluN2A signaling modules and promoted the phosphorylation of Src and GluN2A

**Figure 4:**
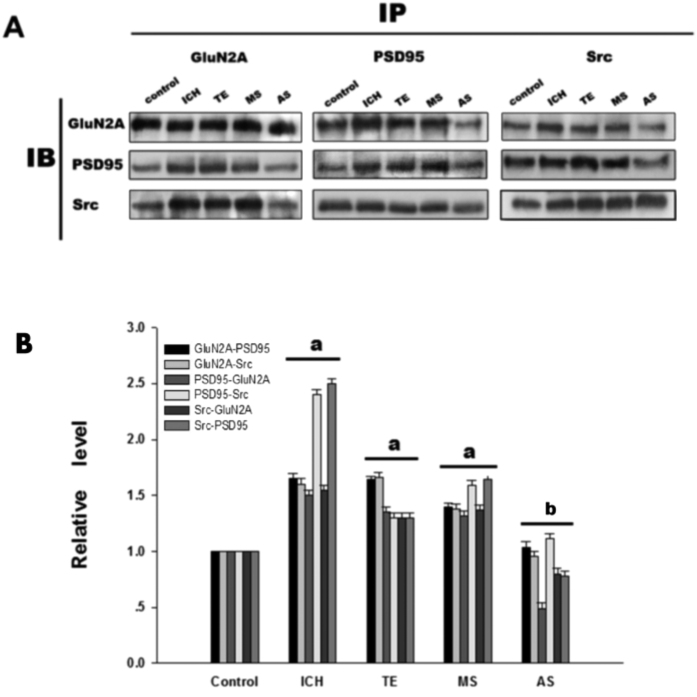
ICH enhanced the interactions among Src, PSD95 and GluNR2A in rat caudate putamen (CPu). (**A**) Reciprocal co-IP analysis of interactions of GluNR2A and PSD95 with Src. Sample proteins were IP with anti-GluNR2A or anti-PSD95 or anti- Src antibodies and then IB with anti-GluNR2A or anti-PSD95 or anti-Src antibody in control, ICH, TE, MS and AS groups. β-actin was used as a loading control. (**B**) Statistics for the interactions in each group. The results are shown as mean ± S.D (n = 5). ^a^*P* < 0.05 vs. Control, ^b^*P* < 0.05 vs. ICH 3 h treatment group.

**Figure 5:**
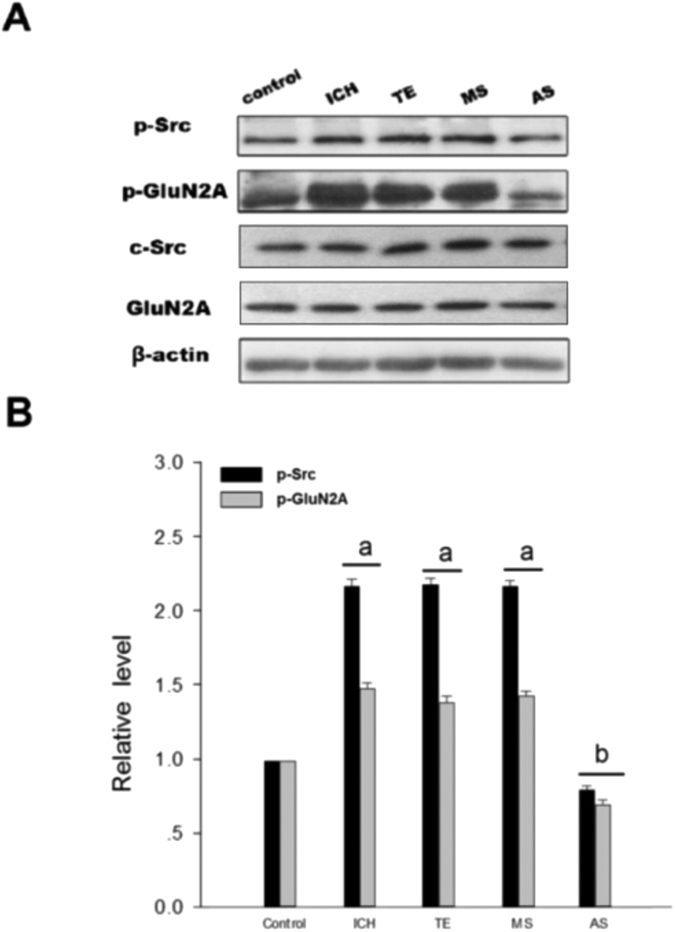
AS-PSD95 and Src antagonist PP2 reduced the tyrosine phosphorylation of Src, GluNR2A after rat ICH in CPu. (**A**) The protein levels of Src, p-Src and GluNR2A were determined by western blot analyses. p-GluNR2A was examined by immunoprecipitation with anti-p-Y antibody followed by immunoblotting with anti-GluNR2A antibody. (**B**) Statistic for p-Src and p-GluN2A in control, ICH, TE, MS and AS groups. The results are shown as mean ± S.D (n = 5). ^a^*P* < 0.05 vs. Control group, ^b^*P* < 0.05 vs. ICH group.

To further investigate the interactions among components of the Src-PSD95-GluN2A module, three kinds of protein immunoprecipitation (IP) and Immunoblotting (IB) were conducted. Previously we found that interactions among Src, PSD95 and GluN2A reached maximum after 3 h of ICH. Among the six time points after ICH including 0.5 h, 1 h, 3 h, 6 h, 12 h, 24 h, 3 h and 6 h time points showed significant differences (data not here). Present results showed that antisense oligonucleotide against PSD95 (AS-PSD95) inhibited the expression of PSD95 and reduced the interactions between Src-PSD95, Src-GluN2A, and PSD95-GluN2A, while the MS-PSD95 could not do so (Fig. 4A,B). In addition, the phosphorylation of Src and GluN2A was significantly increased when compared with the control rats, while AS-PSD95 or PP2 reduced p-Src or p-GluN2A in rats after 3 h of ICH induction (Figs 5A,B and 2B,C).

In COS7 cells, treatment of thrombin (5 U/ml, to mimic the rats ICH model) enhanced the interaction between PSD95 and GluN2A when compared with the control cells; treatment with PP2 reduced PSD95-GluN2A interaction, while co-application of PP2 and SFLLR eliminated the inhibitory effect of PP2 (Fig. 3A,B). Both *in vivo* and *in vitro* results suggested that ICH enhanced the assembly of the Src-PSD95-GluN2A signaling modules and promoted the phosphorylation of Src and GluN2A.

### Enhanced interaction of Src-PSD95-GluN2A and phosphorylation of Src and GluN2A after ICH were PAR1 dependent

**Figure 6:**
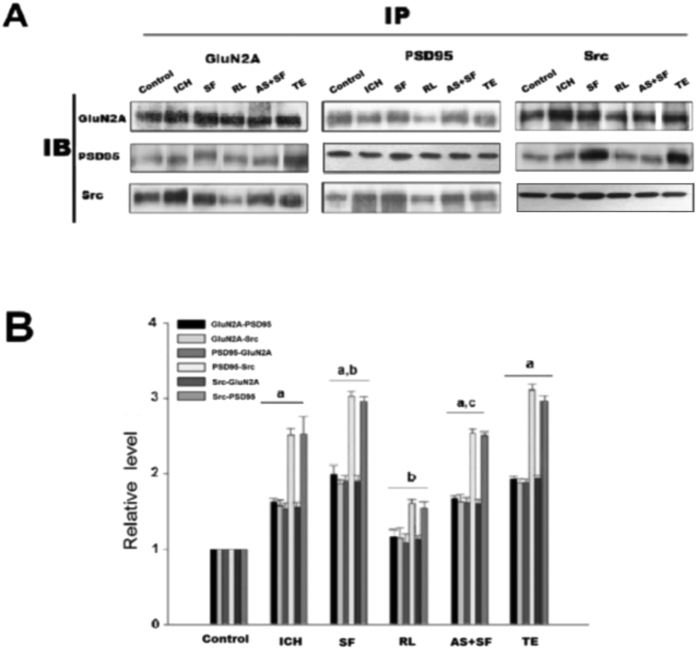
PAR1 enhanced interactions among GluNR2A, PSD95 and Src after rat ICH in CPu. (**A**) Reciprocal co-IP analysis of interactions of GluNR2A and PSD95 with Src. Sample proteins were immunoprecipitated with anti-GluNR2A or anti-PSD95 or anti-Src antibodies and then blotted with anti-GluNR2A or anti-PSD95 or anti-Src antibody. (**B**) Statistics for the interactions in control, ICH, SF, RL, AS + SF and TE groups. The results are given as mean ± S.D (n = 5). ^a^*P* < 0.05 vs. Control, ^b^*P* < 0.05 vs. ICH 3 h treatment group, ^c^*P* < 0.05 vs. RL-treatment group.

Here we showed that treatment of the rats with the PAR1 agonist SF could increase the interaction among Src-PSD95, Src-GluN2A, and PSD95-GluN2A, while treatment of PAR1 antagonist RL significantly reduced the interaction compared with SF group (Fig. 6A,B). To further illustrate whether enhanced interaction of Src-PSD95-GluN2A after ICH were dependent on PAR1, co-application of SF and AS-PSD95 was adopted, and the results showed that AS-PDS95 could counteract the effects of SF. The results indicate that PAR1 mediated the interaction of Src-PSD95-GluN2A molecules after rat ICH. Similar results were also obtained in thrombin (5 U/ml) treated COS7 cells when co-application of SF and PP2 were used (Fig. 3A,B). Collectively, these results indicate that assembly of Src-PSD95-GluN2A signaling molecules after ICH was closely related to PAR1 activation after rat ICH.

**Figure 7:**
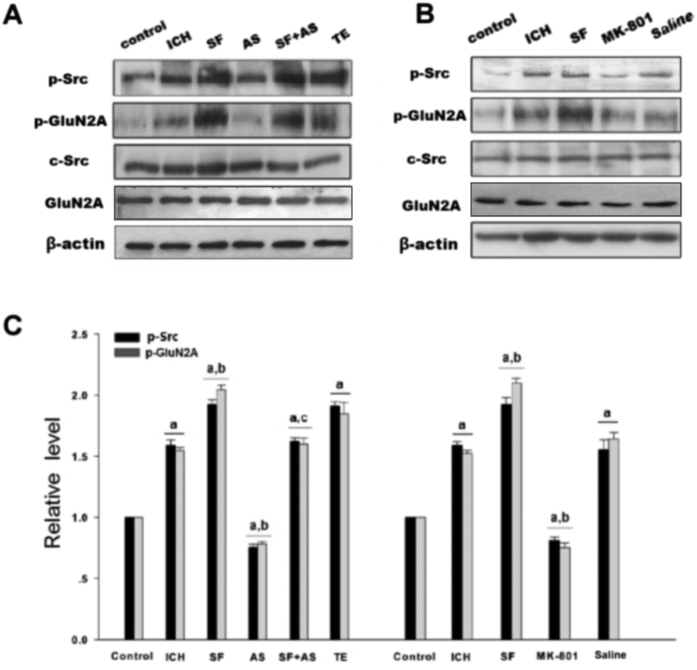
PAR1 increased phosphorylation of Src and GluN2A after rat ICH in CPu. (**A,B**) Western blot analysis for Src, p-Src and GluNR2A protein levels after SF, AS-PSD95, SF + AS-PSD95 and MK-801 treatment. p-GluNR2A was examined by immunoprecipitation with anti-p-Y antibody followed by immunoblotting with anti-GluNR2A antibody. (**C**) Statistics for p-Src, p-GluNR2A, Src and GluNR2A in control, ICH, SF, AS + SF, TE, MK801 and saline groups. The results are shown as mean ± S.D (n = 5). ^a^*P* < 0.05 vs. Control, ^b^*P* < 0.05 vs. ICH 3 h treatment group, ^c^*P* < 0.05 vs. AS group.

In accordance, treatment of AS-PSD95 or MK801 in SD rats reduced GluN2A and Src phosphorylation, while co-application of SF and AS-PSD95 could abolish the effect of SF (Fig. 7A–C). These results indicated that PAR1 mediated GluN2A and Src phosphorylation after ICH.

## Discussion

The major finding of this study is that PAR1 enhanced Src-PSD95-GluN2A signaling molecules interaction through phosphorylation of Src, thereby leading to the activation of NMDA receptor under ICH condition.

ICH enhanced thrombin release and mediated PAR1 activation. Hematoma mass effect is a major cause of cerebral ischemic and edema in ICH^11^,^12^. The level of thrombin generated after ICH was positively correlated with the degree of edema surrounding the hematoma tissue, suggesting that the release of thrombin could be an important factor for brain injuries after hemorrhage^29^,^30^. As a first identified receptor of thrombin, PAR1 is one of the most important PARs involved in neurotoxicity and neuronal apoptosis^31^,^32^. Studies showed that excess thrombin could induce neurotoxic effects and neuronal apoptosis via PAR1 activation^33^,^34^,^35^,^36^. Based on these lines of evidence, we speculated that thrombin directly activated PAR1 in the course of ICH, leading to the activation of a series of downstream signaling pathways that mediate ICH injuries. For induction of ICH, we injected the 50 μl tail blood directly into the rat caudate putamen so that prothrombin could be transformed into thrombin during the process of the coagulation.

Src family is a class of non-receptor tyrosine kinase. Src was not only a cytoplasmic effector enzyme of G protein-coupled receptor PAR1^37^,^38^, but also a functional enzyme regulating ion channel NMDAR^18^,^19^,^20^. Therefore, Src might be an important bridge linking G protein-coupled receptors (PAR1) and NMDAR. In the central nerve systems, NMDAR was the first identified ionotropic glutamate receptor regulated by Src family kinase^20^. Electrophysiological analysis showed that phosphorylation/dephosphorylation of tyrosine controlled the electronic current of NMDAR. NMDAR is co-regulated by two classes of enzymes: PTK and PTP^39^. Inhibiting PTP or inducing PTK activation could increase the electronic current of NMDAR^40^. Src belongs to PTK, which can enhance the function of NMDAR^18^ and affect the current flux through native NMDAR channels^41^. We previously showed that excessive release of glutamate during cerebral ischemia/reperfusion injury caused influx of Na^+^ and Ca^2+^ through NMDAR, and activated Src kinases mediated GluN2A and 2B tyrosine phosphorylation, subsequently increased the activity of NMDAR^19^,^42^,^43^. Herein, we hypothesized that thrombin could activate Src through G protein-coupled receptor PAR1, subsequently Src leads to activation of NMDAR. In this study, we found that PAR1 activator SF significantly increased NMDAR-mediated fEPSP, while antagonist RL reduced f EPSP (Fig. 1A–C). To further investigate PAR1 up-regulated NMDAR activity via Src kinase, co-application of SF and Src inhibitor PP2, the electrophysiological analysis showed that Src inhibitor PP2 significantly abolished the effects of SF on *I*_NMDA_ (Fig. 1D,E). Such results that SF + PP2 abolished the effects of SF were further confirmed *in vivo* by determining the activation of GluN2A phosphorylation with immunoblotting (Fig. 2A,B) as well as in COS7 cells (Fig. 3A,B). Those results indicate that regulatory effects of PAR1 on NMDAR activity are mediated by Src kinase during ICH. Although a broad spectrum inhibitor PP2 of the Src kinase (not the specific Src inhibitor) was used in this study, GluN2A might be tyr-phosphorylated by Fyn and there may be other src-family tyrosine kinases such as Lck/Fyn in the IPed (immunoprecipitated) complex that are inhibited by PP2^44^, Src kinase should be an important occurring^18^,^19^,^20^. It is possible that PAR1 could regulate the Src-mediated activation of NMDAR, and therefore increased glutamate-mediated neuronal death^45^,^46^.

**Figure 8:**
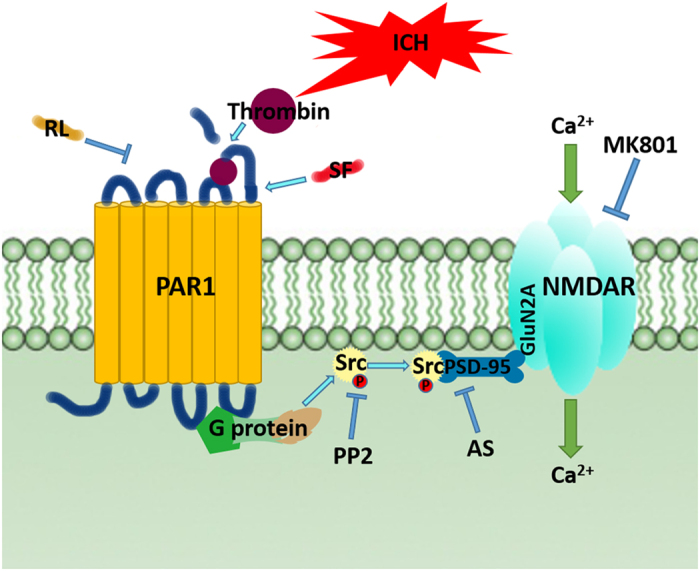
Possible mechanisms for PAR1modulating NMDA receptor. Diagram summarizing our findings that during ICH, activated PAR1 increased the Src activation and subsequently enhanced the interactions among the Src-PSD95-NR2A signaling molecules, which promoted phosphorylation of NMDAR and activation.

In the glutamatergic excitatory post-synaptic density (PSD), receptor proteins, cytoskeletal proteins, and various signaling complex molecules (including protein kinase, phosphatase) could directly or indirectly bind to NMDAR reversibly, forming a dynamic signaling complex with NMDAR as the core^47^,^48^. PSD95 contained three N-terminal PDZ domains (PDZ1, PDZ2, PDZ3), one SH3 domain and one GK domain at the C-terminus. PSD95 could bind to GluN2 subunits (GluN2A and GluN2B) of NMDAR via PDZ2^49^. PSD95 bond SH2 of SrcPTKs through its PDZ3 domain^50^. During cerebral ischemic injury, SrcPTKs and NMDAR substrate GluN2A were recruited to PSD, forming Src-PSD95-GluN2A signaling modules^19^. Formation of this signaling module could firmly anchor SrcPTKs to the NMDAR, resulting in Src close GluN2A and enhance GluN2A tyrosine phosphorylation^51^,^52^. Up-regulation of NMDAR functions thereby aggravates the injuries in excitatory neurons^53^,^54^. In the present study, our results showed that the assembly of the Src-PSD95-GluN2A signaling module, and phosphorylation of Src and GluN2A were enhanced under ICH *in vivo* (Figs 2A,B and 4A,B). AS-PSD95, Src inhibitor PP2, NMDA receptor antagonist MK801 or the PAR1 antagonist peptide RL inhibited the interaction Src-PSD95, Src- GluN2A, and PSD95-GluN2A (Figs 2A,B and 3A,B) and p-Src, p-GluN2A (Figs 2B,C, 3A,B and 7A,B), PAR1 activator SFLLR enhanced such interaction and p-Src, p- GluN2A, while co-application of SF + AS-PSD95, SF + PP2 neutralized the increase of SF (Figs 2A, 3A,B and 6A,B), indicating that GluN2A-PSD95-Src signaling cascades were regulated by PAR1 (Fig. 6). All these results support the hypothesis that PAR1 induces Src activation. Src is a cytoplasmic effector enzyme of G protein-coupled receptor PAR1 which could enhance the interactions among the Src-PSD95-GluN2A signaling modules and enhance the anchor of SrcPTKs to the NMDAR, subsequently inducing tyrosine phosphorylation of GluN2A (Fig. 8). High amount of Ca^2+^ may enter the postsynaptic pyramidal cells through the NMDA receptors, which further activates Src^18^. This positive feedback mechanism may result in the delayed neuronal death after ICH.

In summary, our results demonstrated that during ICH, activated G protein-coupled receptor PAR1 mediated activation of its effector enzyme Src. Activated Src enhanced the interactions among the Src-PSD95-GluN2A signaling molecules and promoted phosphorylation of NMDAR (Fig. 8). By studying this signaling pathway (thrombin → PAR1 → Src-PSD95-GluN2A signal modules → GluN2A), we expect to further explore the role of Src kinase and NMDA receptors in ICH brain injuries, and provide new strategies and drug targets for the treatment of hemorrhagic brain injury.

## Additional Information

**How to cite this article**: Duan, Z.-Z. *et al*. Protease activated receptor 1 (PAR1) enhances Src-mediated tyrosine phosphorylation of NMDA receptor in intracerebral hemorrhage (ICH). *Sci. Rep*. **6**, 29246; doi: 10.1038/srep29246 (2016).
